# The effect of newer anti-rheumatic drugs on osteogenic cell proliferation: an in-vitro study

**DOI:** 10.1186/1749-799X-4-17

**Published:** 2009-05-26

**Authors:** Ajay Malviya, Jan Herman Kuiper, Nilesh Makwana, Patrick Laing, Brian Ashton

**Affiliations:** 1 Wansbeck General Hospital, Woodhorne Lane, Ashington, Northumberland, NE63 9JJ, UK; 2 Joint Reconstruction Unit, Robert Jones and Agnes Hunt Orthopaedic Hospital, Oswestry, SY10 7AG, UK; 3 Orthopaedic Department, Robert Jones and Agnes Hunt Orthopaedic Hospital, Oswestry, SY10 7AG, UK; 4 Arthritis Research Centre, Robert Jones and Agnes Hunt Orthopaedic Hospital, Oswestry, SY10 7AG, UK

## Abstract

**Background:**

Disease modifying anti-rheumatic drugs (DMARDs) may interfere with bone healing. Previous studies give conflicting advice regarding discontinuation of these drugs in the peri-operative setting. No consensus exists in current practice especially with the newer DMARDs such as Leflunomide, Etanercept, and Infliximab. The aim of this study was to assess the in-vitro effect of these drugs alone and in relevant clinical combinations on Osteoblast activity.

**Methods:**

Osteoblasts were cultured from femoral heads obtained from five young otherwise healthy patients undergoing total hip replacement. The cells were cultured using techniques that have been previously described. A full factorial design was used to set up the experiment on samples obtained from the five donors. Normal therapeutic concentrations of the various DMARDs were added alone and in combination to the media. The cell proliferation was estimated after two weeks using spectrophotometric technique using Roche Cell proliferation Kit. Multilevel regression analysis was used to estimate which drugs or combination of drugs significantly affected cell proliferation.

**Results:**

Infliximab and Leflunomide had an overall significant inhibitory effect (p < 0.05). Dexamethasone had a small stimulatory effect that was however strongly donor-dependent. The cox-2 inhibitor Etoricoxib was found to negate or increase the action of two other drugs (Leflunomide and Dexamethasone). Methotrexate and Etanercept had no discernable donor-dependant or donor-independent effect on osteoblast proliferation.

**Conclusion:**

Our study indicates that in-vitro osteoblast proliferation can be inhibited by the presence of certain DMARDs. Combinations of drugs had an influence and could negate the action of a drug on osteoblast proliferation. The response to drugs may be donor-dependent.

## Introduction

Patients with various rheumatologic and inflammatory disease states commonly require drugs for adequate control of their condition. It is well recognized that certain anti-rheumatic drugs may affect inflammation and local immune responses, which are necessary for proper wound healing in the peri-operative setting, thereby potentially resulting in undesirable postoperative complications[1].

Normal bone healing is an essential requisite in the appropriate management of patients with inflammatory arthritis who have fractures or have undergone fusion procedures. It has been anecdotal that, despite good surgery, the results are not always satisfactory. This is possibly due to altered bone healing as a result of the disease process and/or the drugs being used for treatment. It has been argued that these effects might be patient-specific[2]. There is of course the risk of inflammatory flare up on withholding these drugs and this may itself increase the risk of complications[3]. Clinicians must, therefore, carefully evaluate individual patient risk factors, disease severity and the pharmacokinetics of available therapies when weighing the risks and benefits of discontinuing therapy in the peri-operative setting[1].

Several studies, mostly in vitro studies or animal experiments, have been carried out to study the effects of Disease Modifying Anti-Rheumatic Drugs (DMARDs) on bone healing [4-7]. These studies have demonstrated an inhibition of osteoblast proliferation in-vitro in the presence of Methotrexate with no significant effect on osteoblast differentiation. The effect has been shown to be dose dependent [4-7] with some proposing that, at therapeutic dose used in treatment of rheumatoid arthritis, there is no significant effect[6].

Nonsteroidal anti-inflammatory drugs (NSAIDS) are also used in the treatment of rheumatoid arthritis, alone or in combination with DMARDs. It has been shown these adversely effect bone healing [8-13] and this may be more profound during early stages of fracture repair[14]. However, it has been thought that short term use of NSAIDS inhibitors would not produce any deleterious effect because their effect is reversible and dose dependent[12,15,16]. It is acknowledged that these drugs should be used with caution in all patients following osseous trauma, particularly after injuries that may predispose a fracture to delayed union due to osseous, vascular or patient-related factors[11].

There is very little knowledge regarding the effect on bone healing of the newer anti-rheumatic drugs such as Leflunomide and anti-TNF drugs like Etanercept and Infliximab. It is well recognised that patients with severe disease that requires surgery normally receive a combination of drugs. The effect of these drugs in combination has again not been looked into. The need for more data on the perioperative use of DMARDs and biological agents to formulate appropriate guidelines has been established in some reviews[17,18].

In light of these many uncertainties, we carried out an in-vitro study of the effect of various antirheumatoid drugs alone and in combination on the proliferation of osteogenic cells derived from the trabecular bone of resected femoral heads. Uniquely, we looked into the effect of Leflunomide, Etanercept and Infliximab and also various clinically relevant combinations of these drugs. Specifically, we tested the following three hypotheses: (I) these drugs affect osteogenic cell proliferation, (II) their effects on cell proliferation are patient-specific, and (III) these drugs enhance or reduce each other's effects when combined.

## Methods

### General study design

A full factorial design of Experiments approach was used to generate a scheme of drug combinations added to the medium in which trabecular bone fragments from 5 individual donors were grown. The number of osteogenic cells generated during a 14 day culture period was the outcome variable.

### Drugs

The effects of six different drugs were investigated (Table 1). The six drugs were divided in three groups, based on the clinical likelihood of being used in combination. A drug from any group could be combined with a drug from another group, but not with a drug from the same group. In other words, the four drugs from group 3 were not combined.

**Table 1:** The six drugs tested.

	Drug	Concentration (μg/ml or nM)
Group 1	COX-2 inhibitor – Etoricoxib	4 μg/ml (1.1·10^-5 ^M)
Group 2	Methotrexate	1 μg/ml (2.2·10^-6 ^M)
Group 3	Dexamethasone	4 ng/ml
	Etanercept (anti-TNF; rh soluble TNF-receptor)	1 μg/ml
	Infliximab (anti-TNF; chimeric monoclonal antibody)	118 μg/ml
	Leflunomide (pyrimidine synthesis inhibitor)	4 μg/ml (1.5·10^-5 ^M)

The drugs were combined between groups, but not within groups

Samples of pure drugs were obtained directly from the manufacturers and stable solutions were prepared according to the manufacturers instructions. Therapeutic concentrations of the drugs were added to tissue culture medium from Day 1 in various combinations.

### Trabecular bone samples

With approval of the Local Research Ethics Committee, trabecular bone specimens were collected from femoral heads of five relatively young (50–60 yrs) consented donors undergoing total hip replacement for indications other than inflammatory arthritis and who had not been prescribed any of the drugs under study.

### Culture Methods

Osteoblast-like cells were isolated and cultured from trabecular bone of femoral head, as has been described previously [19-21]. Briefly, the trabecular bone was blotted dry and pulled into fragments 2–3 mm in each dimension using forceps. These were washed with several changes of tissue culture medium (α-MEM with Earle's salt and L-Glutamine – Gibco, Paisley, Scotland) to remove cells remaining in the intra-trabecular spaces and plated at 1 fragment per well in a 24-well plate containing 1 ml of control medium (α-MEM containing 1% Gentamicin v/v and 10% foetal bovine serum) or medium supplemented with drugs. Cultures were maintained at 37°C in a humidified atmosphere of 5% (v/v) CO_2 _in air.

The medium was changed at days 3, 7, 10 and 14. The cultures were inspected at days 7 and 14. At day 14 cell number in the culture wells was assessed colorimetrically using the Roche Cell proliferation Kit II (Roche, Mannheim, Germany). This method is as sensitive as radioactive assay with a significantly lower inter- and intra-tester variability [22]. The change in absorbance at 450 nm after 24 h was used for analysis.

### Experimental design and statistical analysis

Each drug was administered at either zero or full therapeutic dose, thus 20 dose combinations could be generated (Table 2). Following the principles of statistical experimental design, each dose combination was added to samples from each of the five femoral head donors. Such an experimental design testing all combinations is known as a "full factorial design", and allows to test the effect on proliferation of each drug separately, and in combination with other drugs [23,24]. The combination with no drug (Mix 1, Table 2) served as control, and was tested an extra five times to assess the standard error. Hence, a total of 25 replicates were tested for each individual donor. In this way, we could test the effects of the drugs on cell number for each donor.

**Table 2:** Drug combinations prepared.

Mix	Etoricoxib	Methotrexate	Dexamethasone, Etanercept, Infliximab or Leflunomide
1	0	0	0
2	0	100%	0
3	100%	0	0
4	100%	100%	0
5	0	0	100% of either
6	0	100%	100% of either
7	100%	0	100% of either
8	100%	100%	100% of either

The four mixes 5–8 exist for all four drugs from group 3, making 16 combinations. Together with the four mixes 1–4, 20 different combinations are possible, and were all prepared.

The results were analysed using multilevel or hierarchical regression analysis. This method takes into account the hierarchical nature of the data, in this case the fact that a number of separate experiments were performed on cells from individual donors [25,26]. The analysis gives simultaneous estimates of significant effects that are constant for all donors (known as "fixed effects" or "constant effects") and significant effects that vary between individual donors (known as "random effects" or "varying effects"). It thus gives a robust framework to disentangle responses that are donor-independent and those that differ between donors. Stepwise multiple regression analysis was used to identify the best predictors of osteoblast number [23,24]. In identifying drugs or their combinations that had a significant effect we used the "effect heredity principle", which states that an interaction term can only be included if at least one of its corresponding main effects is significant[24]. Using this principle, we added and removed constant and varying factors until convergence was obtained, using the likelihood ratio test to compare the various multilevel models[25,26]. A p-value of 0.05 was used to determine whether more complicated models were significantly better than less complicated ones. All statistical analysis was performed with MLwiN vs. 2.10b (Centre for Multilevel Modelling, University of Bristol, UK).

## Results

**Figure 1:**
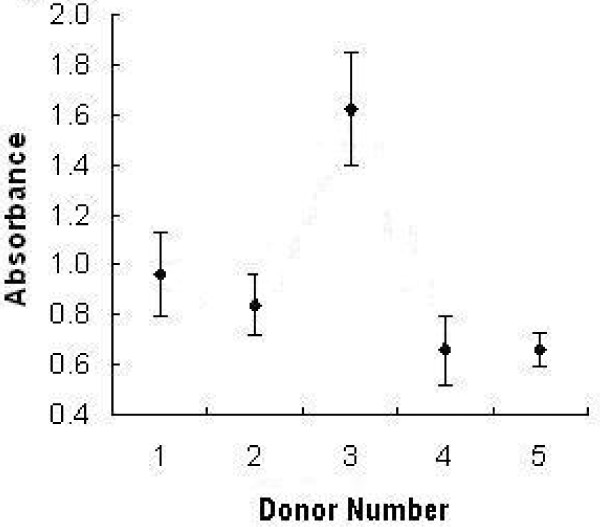
**Average absorbance of the six control samples for each donor**. The difference in absorbance between the donors was significant. (error bar = 1 SEM).

Osteogenic cells were derived from all donors in all drug combinations tested, although there were differences between donors in cell numbers as measured by change in absorbance (p < 0.001 (ANOVA); Figure 1). For this reason, we normalised the results for each donor by the average absorbance of the six control samples cultured with no drugs.

### Drugs with a constant (donor-independent) effect

**Figure 2:**
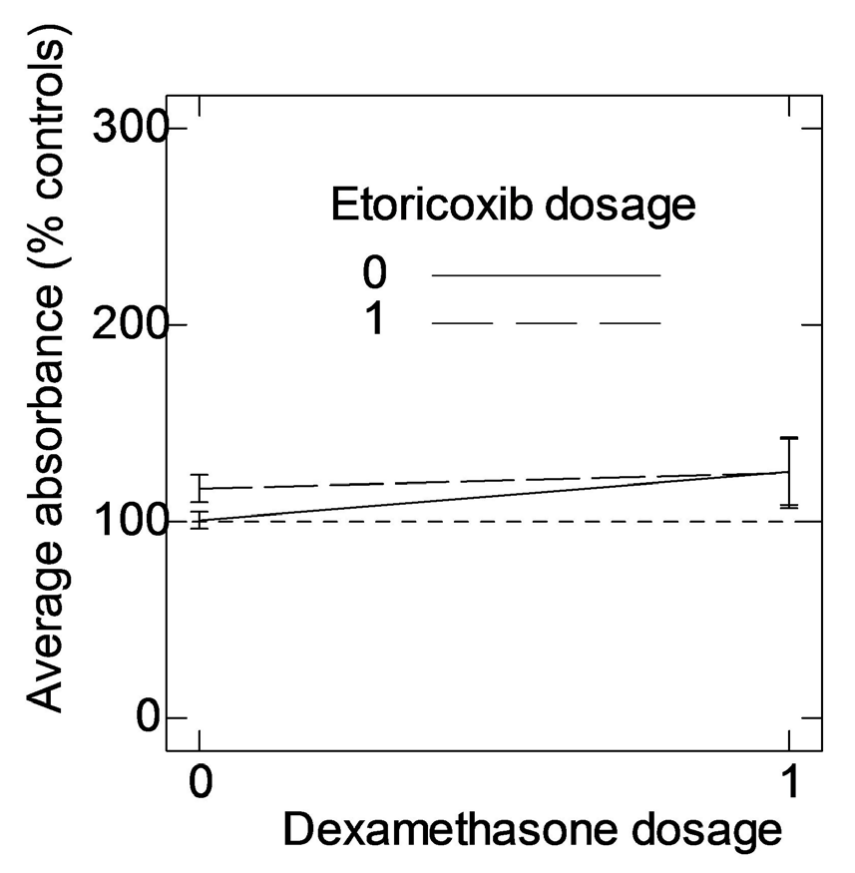
**Interaction between drugs**. On average, Dexamethasone stimulates osteoblast proliferation but in the presence of Etoricoxib this stimulating effect has disappeared. (error bar = 1 SEM).

Two drugs (Infliximab and Leflunomide) had a significant constant effect on normalized cell number, in other words they had an identical effect on cell number in all donors. Both these drugs decreased cell number (Table 3). In addition, Etoricoxib and Dexamethasone in combination had a constant negative interaction effect (Table 3 and Figure 2). In this case, administering Etoricoxib negated the effect of Dexamethasone on proliferation.

**Table 3:** Drugs with constant (patient-independent) effects

Predictor	% change in absorbance per drug dose (SEM)	p-value
Infliximab	-20% (9%)	0.025
Leflunomide	-21% (9%)	0.022
Etoricoxib × Dexamethasone	-14% (7%)	0.041

### Drugs with a varying (donor-dependent) effect

One drug (Dexamethasone) had a significant varying effect on normalized cell number, in other words its effect was significant but differed between donors (Table 4). On average, Dexamethasone increased proliferation (albeit that this effect was negated when Etoricoxib was also administered, Figure 2). There was however a large variation in its effect to the extent that Dexamethasone decreased proliferation in some donors (Table 4). In addition, Etoricoxib and Leflunomide had a significant varying interaction effect (Table 4). On average, the interaction effect was negative (administering Etoricoxib increased the negative effect of Leflunomide on proliferation). There was however a large variation between donors, and in some donors Etoricoxib completely abolished the effect of Leflunomide.

**Table 4:** Drugs with varying (donor-dependent) effects

Predictor	Average effect (SEM)	Between-donors SD of effect
Dexamethasone	8% (12%)	17%
Leflunomide × Etoricoxib	-8% (7%)	4%

### Drugs with no effect

Two of the six drugs (Methotrexate and Etanercept) had no discernable donor-dependent or donor-independent effect on cell proliferation.

## Discussion

In this study, we found that antirheumatoid drugs can affect osteogenic cell proliferation. Two antirheumatoid drugs in particular (Infliximab and Leflunomide) significantly decreased average osteoblast proliferation, whereas the selective COX-2 inhibitor Etoricoxib stimulated average osteoblast proliferation. In addition, we found that the effects of these drugs on cell proliferation can vary between donors. Although on average Dexamethasone increased cell proliferation, its effect varied strongly between donors to the extent that it significantly decreased osteoblast proliferation in some donors. Finally, we found that these drugs can enhance or reduce each other's effects when combined. The COX-2 inhibitor Etoricoxib in particular was able to negate the influence of one anti-rheumatic drug (Dexamethasone) and increase the effect of another (Leflunomide) on cell proliferation. Again, these interactions could differ between donors.

Averaged over all donors, two antirheumatoid drugs (Infliximab and Leflunomide) significantly decreased average osteoblast proliferation. Leflunomide is an antiproliferative drug which inhibits de novo pyrimidine ribonucleotide biosynthesis[27]. Its negative effect on osteoblast proliferation found in this study is therefore not unexpected, although to our knowledge the effect of this drug on osteoblast proliferation has not been investigated before. Infliximab is a newer antirheumatoid drug. It is a TNF-α antibody, acting as a TNF-α inhibitor. An earlier study has shown that human osteoblasts grown in serum release TNF-α, which increases cell proliferation but the effects of which can be blocked by adding TNF-α antibody[28]. Our finding that Infliximab reduces proliferation is therefore most likely explained by its neutralizing effect on released TNF-α. In addition, our study also demonstrates that the negative effects of Leflunomide and Infliximab on proliferation are noticeable regardless of the presence of the other drugs we tested.

We also found that the actions of some drugs on osteoblast proliferation varied largely between the five donors in this study. There has always been a belief that the response to these drugs depends on the individual and the unique constituency of every donor. Although it is well recognised that cells derived from different donors exhibits marked variation in their capacity for proliferation[6,29-31], it is assumed that cells of each donor would lose their identity and all cells despite their origin would behave consistently in a similar manner to various agents. Our study suggests this probably is not the case. Cells of different donors seemed to respond differently to the drugs, suggesting the cells retained their individuality. Multilevel (also known as hierarchical or mixed) regression analysis, the technique we used, takes account of the data hierarchy and allowed to determine drugs that have a significant effect which differs significantly between patients. Using this approach, we found that one drug in particular (Dexamethasone) had a small overall stimulatory effect that did however differ strongly between donors. Dexamethasone has a well recognised stimulatory effect on osteoblast proliferation in-vitro[32]. Although our average findings concur with this general picture, we also found the effects were strongly donor-dependent. Most in vitro studies would see donor-dependency as a "nuisance factor" and remove it at the analysis stage by pooling all data. However, a study on the effect of Dexamethasone on cell proliferation of normal human trabecular bone-derived cells (NHBCs) also found strongly donor-dependent effect of this agent [33].

Genetic polymorphism has been reported as a cause for the variable efficacy of and sometimes adverse reaction to DMARDs[34]. Such genetic polymorphisms may also explain the variation in response from the donors to each of the drugs in our study. The use of pharmacogenetics has been advocated to tailor therapy to individual patients[34]. The experimental design method we followed in this study might be developed in another way to tailor drug selection to specific donors.

Finally, we found that these drugs can influence each other's actions. In this study, the COX-2 inhibitor Etoricoxib interacted with two other drugs (Leflunomide and Dexamethasone), increasing the action of the first and negating that of the second. That drugs can interact is not unexpected. Systematically investigating such interactions in vitro is slowly becoming a routine procedure to design combination chemotherapy and has recently begun to design combination antifungal drug treatments [35-38]. Here, we show how such an approach can also be successfully applied to antirheumatoid drugs. Once found, further studies could clarify the precise mechanisms of such interactions.

We found no strong effect of methotrexate on osteoblast proliferation. Several researchers have reported a dose-dependent effect of methotrexate on proliferation of human osteoblast-like cells. Davies et al[7] reported that culturing human osteoblasts for three days in the presence of methotrexate at a concentration 10^-7 ^M or more, reduces their numbers by 20%. Scheven et al. reported a similar dose-dependent effect, with slightly over 20% reductions in cell numbers after four days for doses of 10^-7 ^M and higher[4,39]. Although methotrexate is likely to have affected bone proliferation in our study, any effect was certainly smaller than the effects of the other drugs investigated. Testing several drugs simultaneously, as we did in this study, has the advantage that one can compare the effects of several drugs and quickly home in on the most influential ones.

We also found no overall effect of the COX-2 inhibitor Etoricoxib on osteoblast proliferation, although it did interact with two other drugs. The absence of an effect seems to contradict earlier reports which reported a deleterious effect of COX-2 selective inhibitors on bone healing[8-10,12,14-16]. This deleterious effect has been proposed to be not only due to interference with prostaglandin metabolism but also inhibition of angiogenesis[11]. However, a recent meta-analysis questions the validity of much of this earlier work[27]. The main critique is that most of the earlier studies have concentrated on animal models, which leaves unknown the effect of COX-2 inhibitors on similar processes in humans. It should also be understood that our model did not directly investigate the effect of a COX-2 inhibitor on bone healing but on osteoblast proliferation in vitro. Osteoblast proliferation is only one of the many processes involved in fracture healing.

Our study has the obvious limitations of any *in-vitro *study for this purpose, namely its difficult direct translation to clinical practice. Fracture healing is a complicated cascade of events, involving several cellular responses of which osteoblast proliferation is only one. Moreover, we studied samples from only five donors. Studying more donors would probably have allowed detecting clearer patterns. The main strength of the study lies with the experimental design. Full factorial designs, such as used in this study, allow to test main effects and first and higher-order interaction effects of several independent variables, such as drugs. The method does this by applying the independent variables, drugs in our case, in each possible combination. This closely mimics the normal scenario in a patient with rheumatoid arthritis, who would for example receive not only Methotrexate but also adjuvant drugs such as COX-2 inhibitors. However, COX-2 inhibitors are now under debate following uncertainty over their cardiac safety and a withdrawal of one of the most popular brands (Vioxx – Rofecoxib). In patients who are not responding to Methotrexate, newer anti-rheumatic (Leflunomide, Infliximab and Etanercept) are obvious next options, alone or in combination with Methotrexate. To the best of our knowledge, the effect of these newer drugs on bone healing has not been investigated.

## Conclusion

We have uniquely been able to study the effect of newer anti-rheumatic and combination drug therapy on Osteogenic cell proliferation. Our study indicates that in-vitro osteoblast proliferation can be inhibited by the presence of antirheumatic drugs. In particular Leflunomide and Infliximab had a relatively small but consistent overall inhibitory effect on osteoblast proliferation. Compared to these two drugs, the other four drugs in our study had no significant or consistent effect on cell number. Clinicians may need to consider this information before embarking on orthopaedic procedures in patients on these drugs. The risk of stopping the drugs temporarily should be weighed against the relatively small risk of impaired bone healing. This is more relevant for the newer antirheumatic drugs and those on combination therapy. The effect of these drugs on osteoblast proliferation may well be patient dependent.

## Abbreviations

DMARD: Disease Modifying Anti-Rheumatic Drugs; COX-2: Cycloxygenase – 2.

## Competing interests

The authors declare that they have no competing interests.

## Authors' contributions

AM coordinated the study, carried out the experiments and drafted the manuscript. JK participated in the design of the study, performed the statistical analysis and helped to draft the manuscript. PWL & NM conceived of the study, and participated in its coordination. BA designed the study, supervised the laboratory experiments, and helped to draft the manuscript. All authors read and approved the final manuscript.
